# From Strain to Loads: Development of a Measurement Solution for Wind Turbine Transmission Input Loads during Drivetrain Testing

**DOI:** 10.3390/s23041824

**Published:** 2023-02-06

**Authors:** Eren Bilen, Baher Azzam, Ralf Schelenz, Tim Runkel, Malte Raddatz, Georg Jacobs

**Affiliations:** Center for Wind Power Drives, RWTH Aachen University, 52074 Aachen, Germany

**Keywords:** multivariate data analysis, load sensing, strain measurement, rotor loads, gearbox loads

## Abstract

As wind energy is paving the way for the energy transition from fossil to renewable energy sources, the ongoing trend of increasing the rated power of wind turbines aims to reduce the overall cost of wind energy. The resulting increase in drivetrain loads motivates the need for wind turbine (WT) drivetrain testing in the development phase of critical components such as the WT main gearbox (GB). While several WT system test benches allow for the application of emulated rotor loads in six degrees of freedom (6-DOF), the drivetrain input loads can significantly differ from the GB 6-DOF input loads due to the design of the drivetrain under test. However, currently available load measurement solutions are not capable of sensing GB input loads in 6-DOF. Thus, this work aims to develop a methodology for converging signals from a purposely designed sensor setup and turbine specific design parameters to compute the GB 6-DOF input loads during WT testing. Strain gauges (SG) and accelerometers have been installed on the low-speed shaft (LSS) of a WT drivetrain under test at the 4MW WT system test bench at the Center for Wind Power Drives. Using the data of the aforementioned sensors, a methodology for computing the GB input loads is developed. The methodology is validated through comparison to the applied loads data provided by the aforementioned test bench. The results demonstrate the high promise of the proposed method for estimating the GB input loads during WT drivetrain testing.

## 1. Introduction

The trend towards increasing the rated power in WTs leads to higher input loads, motivating the development of systems to monitor said loads during operation. Load monitoring can be used in order to avoid high maintenance costs significantly [1]. The majority of WT maintenance costs are caused by unplanned maintenance [2]. Preventive maintenance is scheduled, and includes the replacement of components before a fault occurs in the best case [3]. However, this may involve replacing components when it is not necessary, such that the remaining useful time of the replaced component is not exploited [4]. Predictive strategies indicate when components are running a higher risk of failure and proposes their replacement [5]. Therefore, predicting failure is essential to reducing operation and maintenance costs [6]. 

Conventional CMS may include analysis based on measurements performed by a sensor on its own [7]. Nevertheless, this approach can lead to false alarms and critical conditions being missed by the CMS. According to Bangert, a CMS can yield better results if performs judgment based on multivariate analysis. 

Condition monitoring (CM) is a vast concept that can be approached on different levels and from different perspectives [3]. According to [3], one can consider the finest level, i.e., sub-components, but also the coarse level of a wind farm. Another point of view, they state, is the physical impact of the applied CMS. Thereby, sensory and monitoring equipment can involve intrusive and non-intrusive methods. Furthermore, according to them, CMS can be classified into fault-detecting and fault-predicting systems. 

To decide what components should be equipped with a CMS, one can consider the risk of failure and its consequences, e.g., downtime [3]. According to [3], several studies have investigated the reliability of WT components. They concluded that some components, such as the rotor (especially the pitch-control), transmission and power system tend to have higher failure rates than others. GB and transmission component failures usually lead to high downtimes due to complex repair measures involving special equipment such as cranes [8]. Furthermore, the repair is dependent on weather and wave conditions when occurring in offshore WTs, which can lead to higher downtimes [8]. 

Failures are usually predicted on the basis of failure statistics, as cited by [6]. However, statistics-based prediction of failures and the remaining useful life (RUL), respectively, lack precision, especially when design, loads or operating conditions vary [6]. WTs of the same type in a wind farm, for example, have varying load states in the drivetrain, leading to different degrees of degradation, both overall and in the components [9]. Lamba et al. claim that defects in gears can be caused by fluctuating loads [10], a typical phenomenon in wind turbines. Furthermore, the load state of a WT drivetrain is characterized not only by torsional load, but superimposed loads resulting from aerodynamic turbulence, as cited by [1]. According to [6,9], this can result in load spectra that deviate from the design load spectra, leading to faster degradation of the drivetrain components. Thus, according to [1], load monitoring systems are necessary to obtain more information on the WT components’ degradation level and to predict WT failure.

Though today’s CMSs can be used to gain insight into the drivetrain’s condition, they cannot measure GB input loads. According to [11], several studies have been conducted to measure the torque on the LSS [12,13,14,15,16,17]. In [18], torque measurements were categorized into strain gauges, magnetostrictive, surface acoustic wave, piezoelectric, and optical and inductive twist measurements. Moreover, the aforementioned torque measurement systems can be divided into two different methods: direct and indirect [15]. Direct methods use torque transducers that are intrusive and costly, while indirect methods are non-intrusive and more cost-efficient and, therefore, could be an alternative solution, according to [15]. Indirect methods typically utilize surface strain or a twist angle [19]. In [20], an indirect virtual load sensor algorithm for estimating the torque on the low-speed planetary stage was developed. Another indirect load and RUL estimation approach was presented in [21], employing readily available supervisory control and data acquisition (SCADA) data and vibration data of a CMS. Capelle et al. proposed an indirect torque measurement method using an Augmented Extended Kalman filter, a physics-based model and encoder and strain measurements on the LSS and HSS and the GB housing, respectively [22]. In [23], a torque measurement methodology was proposed using both magnetostrictive effect and twist angle measurement. The disadvantages of an indirect torque measurement method include complexities in the SG and electronics installation on the rotating LSS [15]. Furthermore, superimposed forces can decrease the accuracy of the strain, and thus of the load measurement [24]. There is currently no available load measurement solution capable of sensing WT GB input loads in 6-DOF [1,25,26]. Major obstacles to be overcome are installation complexities due to rotating parts as mentioned earlier and to power the sensor setup [27]. Moreover, according to [27], the LSS deformation is small because of its high stiffness, typically leading to a low signal-to-noise ratio.

The IEC 61400-1 standard [28] provides design load cases for the certification of new WT designs in order to get insight into the load situation experienced by the WTs to be expected during operation [29]. The proposed methodology provides investigators unprecedented insight into the load situation within the drivetrain during testing. In the first application of the proposed method, accelerated testing covering the load combinations to be expected during WT operation is applied to the WT drivetrain under test. Therefore, the insight gained using the proposed method can provide more transparency in the load situation experienced by drivetrain components such as the GB during operation. Reducing WT loads is vital to increase the availability of WTs and reduce maintenance and operation costs [30]. Understanding the true effects of loading on the GB and how to prevent failure is needed to create an accurate model that accounts for known failure modes [31]. By allowing designers to monitor drivetrain loads during testing using the proposed method, load situations experienced by drivetrain components such as the GB can be identified, allowing designers to implement and test load mitigation measures and therefore reduce operation and maintenance costs. 

Thus, this investigation aims to provide a methodology for estimating the 6-DOF GB input loads during WT testing on a WT system test bench. Therefore, data from strain gauges and accelerometers obtained via a telemetry-based sensor data acquisition system are employed. In Section 2, the methods are explained. The sensor setup and the methodology are explained in Section 2.1. In Section 2.2, the validation of the measurement solution is performed. In Section 2.3, we elaborate on challenges that could be encountered with the practical implementation of the proposed methodology. Section 3 presents the results of this investigation, which are discussed in Section 4. Section 5 provides the conclusion of the investigation.

## 2. Methodology

**Figure 1:**
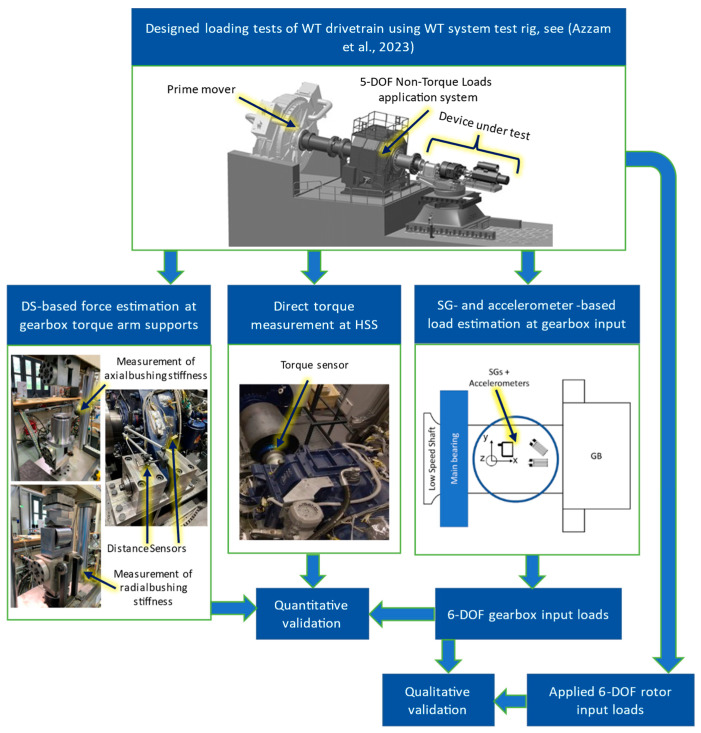
Overview of methodology [29].

The proposed methodology for measuring the 6-DOF WT GB input loads during WT testing is developed and tested using signals from a purpose-designed sensor setup. Sensor data are collected during a measurement campaign on the 4MW WT system test bench at the CWD. Two widely deployed drivetrain configurations [32], 3- and 4-point drivetrain configurations based on the Vestas V52 WT drivetrain, were tested on the test bench during the campaign. The two drivetrains share the same GB, a GPV 306 by Bosch Rexroth. For more details on the design of experiments of the measurement campaign, see [14]. An overview of the methodology is provided in Figure 1. Section 2.1 describes the sensor setup and the implemented methodology to process the resulting signals to measure the GB 6-DOF input loads. Section 2.2 outlines the techniques utilized for validating said load measurements, while Section 2.3 explains considerations and challenges in the practical implementation of the proposed method. 

### 2.1. Sensor Setup and Load Measurement Methodology

**Figure 2:**
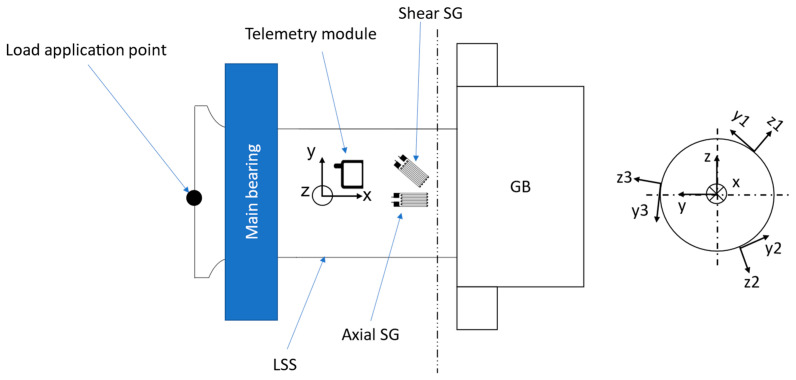
Illustration of LSS with load application point and the telemetry-based sensor system with SGs and their inertial COSs in the cross-section on the right.

In this investigation, strain and acceleration data are employed to compute the respective GB 6-DOF input loads, listed in Table 1, according to the Cartesian coordinate system (COS) shown in Figure 2.

The aforementioned sensor data are collected from three identical sets of sensors, each installed equidistantly 120° apart along the circumference of the LSS. Figure 2 illustrates one such sensor set as mounted on the LSS, where the telemetry module contains a 3-dimensional (3D) accelerometer and transmits the collected signals from the adjacent strain gauge and the accelerometers to a receiver unit during drivetrain testing. As illustrated in the figure, each telemetry transmitter module is mounted on the surface of the LSS. Each module contains a 3D accelerometer with three axes oriented along the illustrated inertial COSs shown to the right of Figure 2. The accelerometer signals in the *y*- and *z*-directions are eventually used in post-processing to determine the angular position of each of the three sensor sets during LSS rotation. Apart from a telemetry module, each one of the abovementioned sensor sets includes two full bridge strain gauges (SG) applied on the LSS surface in parallel to the *x*-axis (hereinafter referred to as axial SG) and rotated 45° with respect to the *x*-axis (hereinafter referred to as shear SG), respectively, as illustrated in Figure 2.

A telemetry-based sensor data acquisition system is necessary due to the rotating points of interest for measuring strain on the surface of the LSS during drivetrain testing and, eventually, to estimate the 6-DOF GB input loads. The voltage signals from the SGs first need to be converted to strain, which is achieved through the conversion outlined in [33,34]. Using the aforementioned strain signals as input, the 6-DOF GB input loads are computed using the fundamental equations of beam theory. Gross et al. provide Equations (1)–(3) to compute the 6-DOF forces and moments on a beam according to the beam theory [35]. To obtain the GB input loads $F_{x_{GB}}$, $M_{y_{GB}}$ and $M_{z_{GB}}$ from the axial SGs measurements, first the equation for the total axial strain of each axial SG resulting from the three loads, i.e., Equation (1) is established [35]. Equation (1) can be used to compute the axial stress that results from an axial force and bending moments around the *y*- and *z*-axis in a beam.

(1) $$\sigma =\frac{F_{N}}{A}+\frac{M_{y}}{I_{y}}z-\frac{M_{z}}{I_{z}}y$$

where $F_{N}$ denotes the normal force along the longitudinal axis, $M_{y}$ and $M_{z}$ are the bending moments around the *y*- and *z*-axes, respectively, $I_{y}$ and $I_{z}$ are the second moment of area with respect to the *y*- and *z*-axes, respectively, A is the cross-section of the beam, and *y* and *z* are the Cartesian coordinates at which the axial stress is calculated.

Equations (2) and (3) are used to compute the shear stresses that result from torsional load and lateral forces, respectively.

(2) $$\tau_{T}=\frac{T}{W_{T}}$$

where T denotes the torsional load around the longitudinal axis of the beam and $W_{T}$ is the torsional section modulus.

(3) $$\tau_{F_{i}}=\frac{F_{i}\;S_{j}}{I_{j}\;b_{i}}$$

where $S_{j}$ is the first moment of area with respect to the *j*-axis, $I_{j}$ is the second moment of area with respect to the *j*-axis, and $b_{i}$ is the width along the *i*-axis.

By employing Hooke’s law, Equation (1) is used to establish Equation (4) in order to obtain the GB input loads $F_{x_{GB}}$, $M_{y_{GB}}$, and $M_{z_{GB}}$.

(4) $$\epsilon_{i}\left(\phi_{i}\right)=\frac{1}{E}\left(\frac{F_{x_{GB}}}{A_{LSS}}+\frac{M_{y_{GB}}\;r_{out}\;cos(\phi_{i})}{I_{y}}+\frac{M_{z_{GB}}\;r_{out}\;sin\left(\phi_{i}\right)}{I_{z}}\right)$$

where $\phi_{i}$ denotes the angular location of each SG, $A_{LSS}$ is the cross-section of the LSS, $r_{out}$ is the outer radius of the LSS, and $I_{y}$ and $I_{z}$ are the second moments of area with respect to the y- and z-axes of the LSS cross-section, respectively. Using accelerometers at the location of the strain measurement, the direction of acceleration at that point relative to the direction of gravity is used to determine the angular position $\phi_{i}$ of the *i*-th sensor set according to Equation (5).

(5) $$\phi_{i}=atan2({acc}_{y_{i}},acc_{z_{i}})$$

where ${acc}_{y_{i}}$ and ${acc}_{z_{i}}$ are the acceleration measurements in the *y*- and *z*-directions, respectively.

Equation (4) is used to establish a system of three equations to compute the three loads $F_{x_{GB}},\;M_{y_{GB}}$ and $M_{z_{GB}}$ employing the set of signals from the three axial SGs leading to the system of equations (SoE) (6).

(6) $$\underset{\epsilon_{ax}}{\underbrace{\left(\begin{array}{c} \epsilon_{1}\\ \epsilon_{2}\\ \epsilon_{3} \end{array}\right)}}=\underset{K_{ax}}{\underbrace{\frac{1}{E}\left(\begin{array}{ccc} 1/A_{LSS} & r_{out}\;cos\left(\phi_{1}\right)/I_{y} & r_{out}\sin\left(\phi_{1}\right)/I_{z}\\ 1/A_{LSS} & r_{out}\;cos\left(\phi_{2}\right)/I_{y} & r_{out}\sin\left(\phi_{2}\right)/I_{z}\\ 1/A_{LSS} & r_{out}\;cos\left(\phi_{3}\right)/I_{y} & r_{out}\sin\left(\phi_{3}\right)/I_{z} \end{array}\right)}}\underset{L_{ax}}{\underbrace{\left(\begin{array}{c} F_{x_{GB}}\\ {M_{y}}_{GB}\\ M_{z_{GB}} \end{array}\right)}}$$

After rearranging the SoE (6), Equation (7) is obtained with the load vector $L_{ax}$ containing the 3-DOF GB input loads $F_{x_{GB}}$, $M_{y_{GB}}$, and $M_{z_{GB}}$.

(7) $$L_{ax}=K_{ax}^{-1}\epsilon_{ax}$$

For the estimates of the GB input loads $T_{x_{GB}}$, $F_{y_{GB}}$ and $F_{z_{GB}}$, first, Equation (8) is established for the total shear stress measured by each of the three shear SGs for the matter at hand by use of Equations (2) and (3). Similarly, Equations (2) and (3) are used to compute the total shear stress, using Equation (8), which is comprised of the shear stress induced by the torsion due to torque $T_{x_{GB}}$, i.e., the first summand, and the shear stress induced by the lateral forces due to $F_{y_{GB}}$ and $F_{z_{GB}}$, i.e., the second and third summands, respectively.

(8) $$\tau_{i}\left(\phi_{i}\right)=-\frac{T_{x_{GB}}}{W_{T}}+\frac{4}{3}\frac{F_{y_{GB}}}{\pi r_{out}^{2}}\cos\phi_{i}-\frac{4}{3}\frac{F_{z_{GB}}}{\pi r_{out}^{2}}\sin\phi_{i}$$

where $W_{T}$ is calculated using Equation (9):

(9) $$W_{T}=\frac{\pi }{2}\frac{{r_{out}}^{4}-{r_{in}}^{4}}{r_{out}}$$

where $r_{in}$ and $r_{out}$ denote the inner radius and outer radius of the cross-section of the hollow LSS at the point of strain measurement, respectively. Equation (8) is used to establish a system of three equations to compute the three loads $T_{x_{GB}}$, $F_{y_{GB}}$ and $F_{z_{GB}}$ employing the set of signals from the three shear SGs leading to the system of equations (SoE) (10).

(10) $$\underset{\tau }{\underbrace{\left(\begin{array}{c} \tau_{1}\\ \tau_{2}\\ \tau_{3} \end{array}\right)}}=\underset{K_{sh}}{\underbrace{\left(\begin{array}{ccc} -\frac{1}{W_{T}} & \frac{4}{3\pi r_{out}^{2}}\cos\phi_{1} & -\frac{4}{3\pi r_{out}^{2}}\sin\phi_{1}\\ -\frac{1}{W_{T}} & \frac{4}{3\pi r_{out}^{2}}\cos\phi_{2} & -\frac{4}{3\pi r_{out}^{2}}\sin\phi_{2}\\ -\frac{1}{W_{T}} & \frac{4}{3\pi r_{out}^{2}}\cos\phi_{3} & -\frac{4}{3\pi r_{out}^{2}}\sin\phi_{3} \end{array}\right)}}\underset{L_{sh}}{\underbrace{\left(\begin{array}{c} T_{x_{GB}}\\ F_{y_{GB}}\\ F_{z_{GB}} \end{array}\right)}}$$

After rearranging the SoE (10), Equation (11) is obtained with the load vector $L_{sh}$ containing the 3-DOF GB input loads $T_{x_{GB}}$, $F_{y_{GB}}$ and $F_{z_{GB}}$.

(11) $$L_{sh}=K_{sh}^{-1}\tau$$

The estimated 6-DOF GB input loads are validated through the methodology explained in the following section.

### 2.2. Validation of Direct Measurement Solution

**Figure 3:**
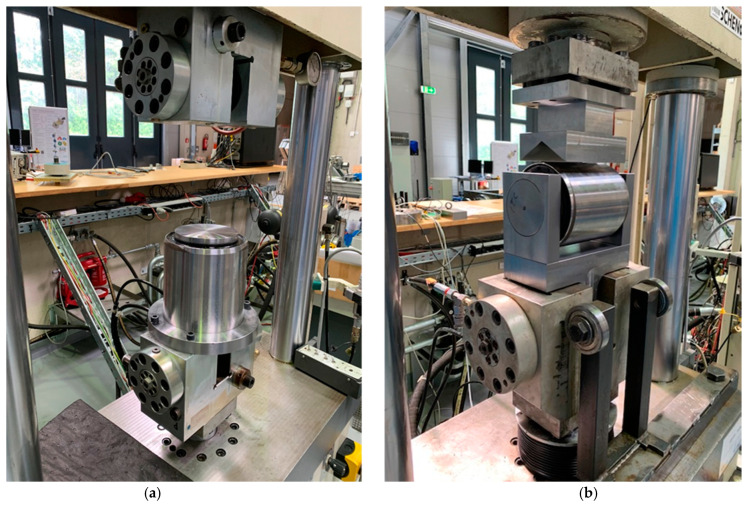
Bushing tests for stiffness estimation in the axial (**a**) and radial (**b**) directions.

Since a solution for measuring 6-DOF GB input loads is not commercially available, sources of validation are limited. Furthermore, due to the design differences between the two tested drivetrains, the 4-point drivetrain non-torque GB input loads are expected to be minuscule compared to those of the three-point drivetrain [31]. Therefore, the methodology for the GB input loads estimation is first applied to a three-point drivetrain in this paper. Nevertheless, the proposed methodology is theoretically applicable to a 4-point drivetrain. Combining domain knowledge of the drivetrain design and knowledge of reference loads from different sensor data acquisition systems, a qualitative validation of the estimated GB input loads can be conducted. Therefore, the loads applied by the load application system of the 4 MW WT system test bench as well as a purposely designed set of DS aiming at measuring the deformation of the torque arm bushings are employed. For a more detailed explanation of the load application system of the test bench, see [18]. The aforementioned DS are applied to each of the left and right GB torque arms to measure their relative displacement with respect to the machine carrier in both the axial and vertical directions. In order to estimate the forces ${F_{x}}_{GB}$ and $F_{z_{GB}}$ from the abovementioned displacements, the stiffness of the torque arms bushings are estimated empirically. For the case of the three-point drivetrain, the axial and radial stiffness of the bushings supporting the GB torque arms is tested using a hydraulic press as shown in Figure 3a,b, respectively. In these tests, the bushings were repeatedly subjected to their rated loads, starting from an unloaded state with frequencies of 0.01, 0.1, and 1 Hz with the resulting deformation measured by the test bench shown in Figure 3. In the manner, the radial and axial stiffness of the bushings were empirically estimated. 

Similarly, the radial stiffness of the torque arm supports of the 4-point drivetrain configuration was empirically estimated in earlier tests conducted at the CWD using the same equipment shown in Figure 3. Hereinafter, force estimates obtained from the aforementioned DS of $F_{x_{GB}}$ and $F_{z_{GB}}$ are referred to as $F_{x_{DS}}$ and $F_{z_{DS}}$, respectively. To summarize, Table 2 provides an overview of the aforementioned loads used to validate the load estimates reached using the proposed method in Section 2.1. The table also provides the respective source of each load as explained in this section. The IDs listed in Table 2 are used hereinafter to refer to each respective load. 

**Figure 4:**
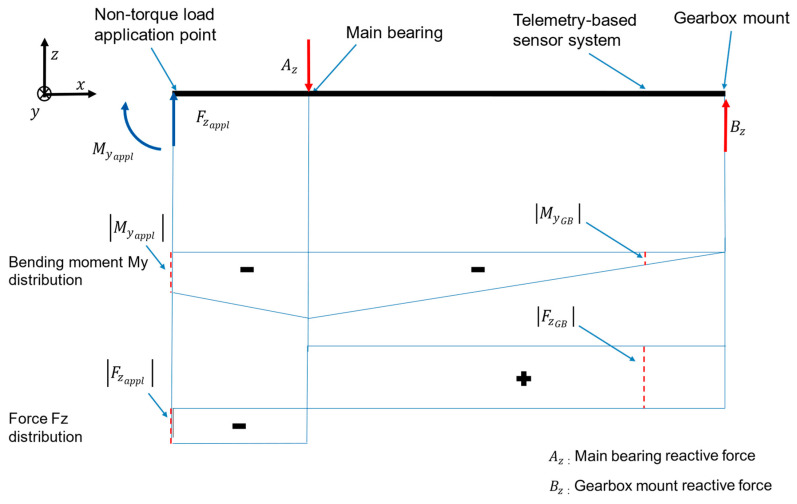
Bending moment around the *y*-axis ($M_{y}$) and force along the *z*-axis $\left(F_{z}\right)$ distributions along the LSS of an example load case with applied bending moment $M_{y_{appl}}$ and applied force $F_{z_{appl}}$.

According to the internal loads theory, the internal loads at the GB input, i.e., the estimated GB input loads (see Section 2.1), are the result of the applied loads [36]. Due to the main bearing arrangement, the non-torque GB input loads differ from the non-torque loads applied by the test bench as illustrated by the red dash-lined loads shown in the exemplary load distribution in Figure 4.

Knowledge about the aforementioned internal loads expected due to the applied loads and the drivetrain design can be used to validate the estimated loads at the GB input qualitatively.

If there is no other torque than the torque applied to the LSS, the estimated GB input torque $T_{x_{GB}}$ is expected to be equal to the applied torque, according to the internal loads theory [36]. Nevertheless, losses due to the drivetrain are expected to lead to a deviation between the estimated and applied torque. As a measure of the performance of the proposed methodology for the torque estimation, the error of the GB input torque $T_{x_{GB_{i}}}$ at each time step in the time series is estimated. Therefore, the *R*^2^ score is calculated using Equation (12) [37]:

(12) $$R^{2}\left(T_{x_{GB_{i}}},T_{x_{HSS_{i}}}\right)=1-\frac{\sum_{i=1}^{n}{\left(T_{x_{GB_{i}}}-\frac{1}{i_{GB}}T_{x_{HSS_{i}}}\right)}^{2}}{\sum_{i=1}^{n}{\left(T_{x_{GB_{i}}}-\frac{1}{i_{GB}}\bar{T_{x_{GB_{i}}}}\right)}^{2}}$$

where $T_{x_{HSS_{i}}}$ is the torque measured on the high-speed shaft (HSS) at each time step, the GB ratio $i_{GB}$ = 61.92 and $\bar{T_{x_{GB_{i}}}}$ the mean value of the GB input torque. The mean value of the GB input torque is computed for each test run, and each test run is 20 min long. The torque on the HSS is measured by using a torque transducer.

Moreover, reactive GB input moments $M_{y_{GB}}$ and $M_{z_{GB}}$ are expected to result from the applied forces $F_{z_{appl}}$ and $F_{y_{appl}}$, respectively, and from applied moments $M_{y_{appl}}$ and $M_{z_{appl}}$, respectively. Reactive GB input forces $F_{x_{GB}}$, $F_{y_{GB}}$ and $F_{z_{GB}}$ are expected to result from applied forces $F_{x_{appl}}$, $F_{y_{appl}}$ and $F_{z_{appl}}$, respectively. Furthermore, the reactive GB input forces $F_{y_{GB}}$ and $F_{z_{GB}}$ can result from the applied moments $M_{z_{appl}}$ and $M_{y_{appl}}$, respectively. The major challenges that need to be considered when the methodology for estimating and validating the GB input loads is applied are explained in the following section. Solutions to tackle these challenges are also provided.

### 2.3. Considerations and Challenges in Practical Implementation

**Figure 5:**
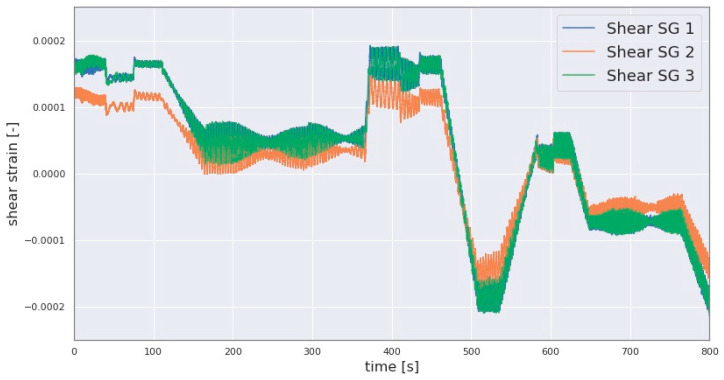
Three shear strain signals including the deviation between the signals.

In this investigation, sensor data from SGs and accelerometers installed on the LSS are used to compute the GB input loads. The data from the abovementioned sensors can include errors such as a deviation in the amplitude of the signals from one of the shear SGs relative to other SGs of the same type, leading to greater error in the computed loads if not mitigated. In this investigation, this error was present in the collected shear strain signals, as demonstrated in Figure 5, where the shear strain signals transmitted from the three telemetry modules are superimposed. In the macro view showing the full range of strain measurement in Figure 5, deviations in the amplitude of the signals are expected to heavily overlap, as is the case for the signals originating from telemetry modules 1 and 3 in the figure. An equidistant phase is also to be expected, since the sensors are equidistantly placed along the circumference of the LSS as explained in Section 2.1. However, one of the aforementioned signals includes an apparent deviation in amplitude and slopes when compared to the other two signals, as can be seen in the difference between the signal from telemetry module 2 and the signals from modules 2 and 3, as shown in Figure 5. Moreover, the orange-colored signal indicates a drift in the signal overtime where its aforementioned amplitude deviation transitions from a negative- to a positive-valued deviation in the sequence of the signal, as shown in Figure 5. In other words, the difference in amplitude between the problematic signal and the other two signals changes over time to range from a negative to a positive difference as demonstrated in Figure 5. The signals shown in Figure 5 resulted from a test run involving subjecting the three-point drivetrain to quasi-static 6-DOF rotor load combinations.

To resolve this error, the Pseudocode in Algorithm 1 is provided. In this investigation, a window length of 6 s was chosen.
**Algorithm 1** For mitigation of the error shown in Figure 5**Input:** Synchronised (Syn.) signal referred to as Correct_signal, Syn. signal referred to as the Incorrect_signal, window_length[sec], sampling_rate[Hz] **Output:** Processed_Signal of the assumed faulty signaltotal_signal_length ← LENGTH(Correct_signal)/sampling_rate n ← CEILING (total_signal_length[sec]/window_length) **SPLIT** Correct_signal into n nonoverlapping windows **SPLIT** Incorrect_signal into n nonoverlapping windows **FOR** each time window DO: Offset ← MEAN(current time window of Correct_signal) − MEAN(current time window of Incorrect_signal) Current time window of Processed_Signal ← Current time window of Incorrect_signal + Offset //Current time window of Processed_Signal is appended to the processed_signal below Processed_Signal ← **STACK**(Current time window of Processed_Signal) **END FOR**

With the pseudocode from Algorithm 1, the aforementioned deviations in the amplitude can be mostly mitigated. However, the implementation of this solution is likely to result in artificial discontinuities in the corrected signal at the boundaries of the windows implemented in the pseudocode from Algorithm 1. This is due to differences between the slopes of the windows of the signal to be corrected and the signal, which is assumed correct. To resolve this error, the pseudocode in Algorithm 2 is implemented, whereby new window boundaries are defined centered around the boundaries of the windows implemented in Algorithm 1. The respective slope of the corrected signal is then calculated for the signal within each window. This is performed by calculating the slope between the first and last data points in a given window. The coordinates of these boundary data points for each window are also stored. The original signal to be corrected (before any correction is applied) is also split into the newly defined windows with the slope of each window calculated using the same approach. Each window of the original signal is then rotated based on the difference between its gradient and the gradient of the corresponding window of the corrected signal (resulting from Algorithm 1) by first identifying the angle between the two gradients using Equation (13).

(13) $$ϴ=-\arctan\left(\frac{m_{original}-m_{corrected}}{1+m_{original}\;m_{corrected}}\right)$$

where $m_{original}$ is the aforementioned slope of the respective signal in a given window from the original signal to be corrected (before any correction is applied) and $m_{corrected}$ is the slope of the respective signal in the corresponding window from the corrected signal (resulting from Algorithm 1).

The signal within a given window from the original signal is then extracted and transformed by Equation (14), where $t_{\varepsilon }$ contains each index in the window and ε the respective strain values, as demonstrated in the pseudocode listed in Algorithm 2.

(14) $$\varepsilon_{rotated}=t_{\varepsilon }\sin ϴ+\varepsilon \cos ϴ$$

**Figure 6:**
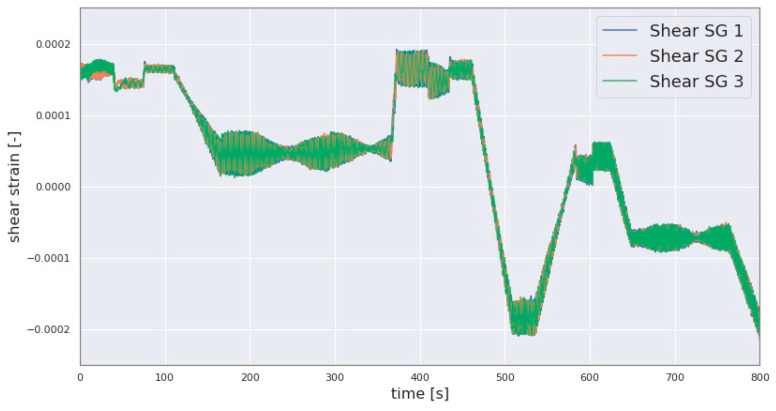
Three shear strain signals after applying the Algorithms 1 and 2.

Each window of the corrected signal resulting from Algorithm 1 is then replaced with the corresponding rotated signal resulting from Equation (14) by translating the latter to the previously stored coordinates of the boundary data points for each window. This procedure alters the original signal only in its gradient and offsets but not in its phase which is necessary for computing the forces $F_{y_{GB}}$ and $F_{z_{GB}}$. The strain signals after applying the algorithms demonstrated in Algorithms 1 and 2 are shown in Figure 6.
**Algorithm 2** For the mitigation of the signal discontinuities resulting from the pseudocode in Algorithm 1**Input:** Processed_Signal (output from Pseudocode in Algorithm 1), Synchr. original signal referred to as Original_signal, window_length[sec], sampling_rate[Hz] **Output:** Processed_Signal (eliminated *jumps*)**FOR** each time window DO: start_idx ← index of the first data point of the current time window stop_idx ← index of the last data point of the current time window $m_{original}$ ← (Original_signal [stop_idx + window_length/2)] − Original_signal [start_idx + window_length/2])/(stop_idx − start_idx) $m_{filtered}$ ← (Processed_Signal [stop_idx] − Processed_Signal [start_idx])/(end_idx − start_idx) theta ← by means of $m_{filtered}$ and $m_{original}$ according to Equation (13) ε ← Original_signal [(start_idx + 0.5 × window_length) to (stop_idx + 0.5 × window_length)] ε ← ε − MEAN(ε) //VECTOR() generates an evenly spaced vector covering the specified range below $t_{\varepsilon }$ ← VECTOR(0 to LENGTH(ε) with increments of 1) − (LENGTH(ε)/2) $\varepsilon_{rotated}$ ← by means of ε, $t_{\varepsilon }$ and theta according to Equation (14) Processed_Signal [start_idx to stop_idx] ← $\varepsilon_{rotated}$ **END FOR**

As is demonstrated with the variations in the amplitudes of the SG signals shown in Figure 5, such lower ranges of amplitude can also result in a variation in the scale of some calculated forces. In such cases, the scale of the calculated loads may be underestimated. However, as shown in Figure 5, the profile of the signal may be correct. Comparisons with the aforementioned sources of validation, such as the DS-based estimated forces, can indicate whether this is the case. They can also be used to correct the scale of the calculated loads by scaling the telemetry-based sensor signals to that of the DS-based sensor signals such as $F_{z_{DS}}$. This scaling operation could also be extended to other similarly affected load estimates by the amplitude deviation.

Furthermore, it is expected that the axially installed SGs may prove ineffective for determining the axial force at the GB input $F_{x_{GB}}$ due to the relatively high axial stiffness of the LSS. Therefore, an alternative approach for estimating the GB input axial force during testing is provided by utilizing the applied force $F_{x_{appl}}$ and the DS measurements-based force $F_{x_{DS}}$. Due to the drivetrain design and the resulting internal load distribution, the ratio between the DS-based axial force $F_{x_{DS}}$ and the applied axial force $F_{x_{appl}}$ is used to estimate the GB input axial force by scaling the aforementioned load $F_{x_{appl}}$. Thereby, $F_{x_{appl}}$ and $F_{x_{DS}}$ are collected from a single-load test scenario involving the sole application of drivetrain input axial force to the three-point drivetrain. During the test, different levels of drivetrain input axial force are reached. The resulting data are used to estimate the contribution of $F_{x_{appl}}$ to $F_{x_{GB}}$ under different levels of $F_{x_{appl}}$ and hence an estimate of the ratio between $F_{x_{appl}}$ and $F_{x_{GB}}$. This ratio is then used to estimate $F_{x_{GB}}$ from $F_{x_{appl}}$ in this alternative method of estimating GB input axial force. 

Moreover, in the case the SG-based GB input forces $F_{y_{GB}}$ and $F_{z_{GB}}$ are not well conditioned, an alternative, hybrid method is proposed in which a scale correction is performed in a single-load test scenario for the drivetrain input force in the *z*-direction using the DS-based approach for the $F_{z_{DS}}$. In addition, if high-frequency noise remains in said forces, these can be filtered using a Savitzky-Golay filter [38]. Therefore, the window length of 2001 sample points at a general sampling rate of f = 500 Hz and a polynomial order of 3 can be chosen as filter parameters.

**Figure 7:**
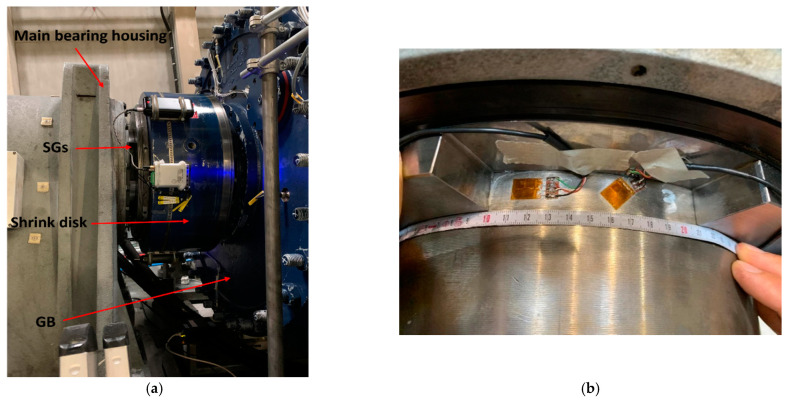
GB input (LSS) of the 4-point drivetrain (**a**) with limited space on the LSS and close-up of the SG location (**b**).

In the 4-point drivetrain configuration, surface at the GB input on the LSS for the SG application can be limited, as shown in Figure 7a. Thus, cutouts are implemented on the sleeve between the main bearing housing and the GB to create surface for the SGs, as can be seen in Figure 7b. However, the sleeve leads to a restriction of the deformation of the LSS resulting in a reduced strain measurement since the SGs are placed in these cutouts. Thus, loads may be underestimated. Therefore, scaling the amplitude between the applied and the estimated torque at the GB input, i.e., $\frac{T_{x_{appl}}}{T_{x_{GB}}}$, can be used for a better approximation of the GB input torque $T_{x_{GB}}$. The following section provides the results obtained when using this methodology to estimate the GB input loads after addressing the challenges described in this section. 

## 3. Results

**Figure 8:**
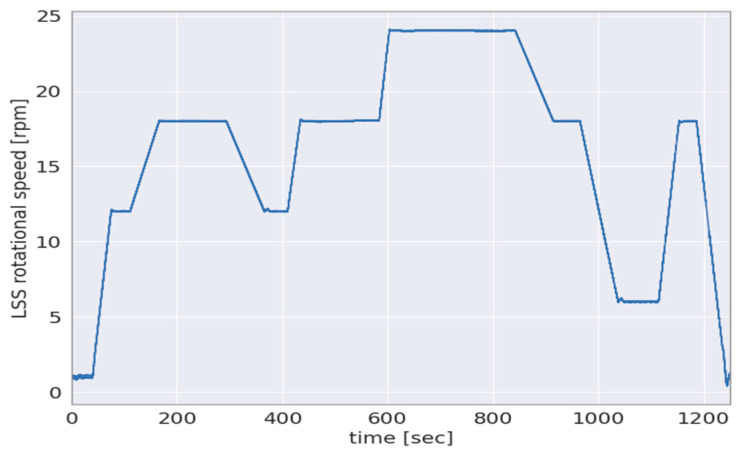
Rotational speed in rpm of the LSS in multiple-load test scenario.

The GB input loads in the 6-DOF are estimated based on strain and accelerometer data according to the methodology outlined in Section 2.1. Furthermore, for qualitative validation of the aforementioned loads, the loads applied by the load application system of the 4MW WT system test bench, the data of a torque transducer at the HSS and the estimated loads of a DS-based sensor solution are employed, as explained in Section 2.2. The first results of the proposed method for GB input 6-DOF loads estimation are provided for a three-point drivetrain in this section. Two test scenarios are investigated for the abovementioned validation of the 6-DOF GB input loads. The rotational speed of the LSS is shown in the multiple-load test scenario in rotations per minute (rpm) in Figure 8.

**Figure 9:**
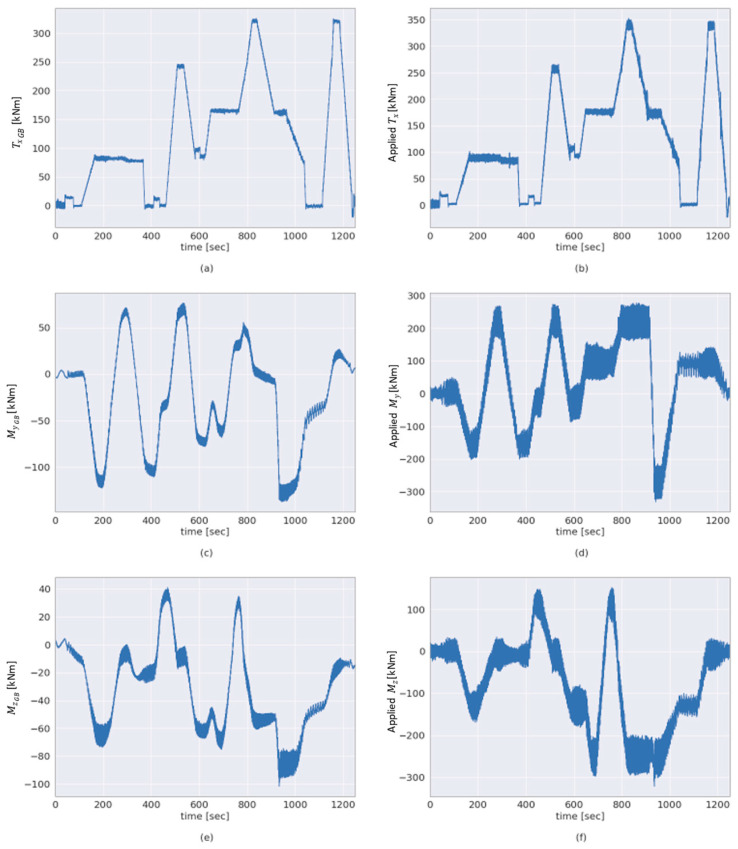
Comparison between estimated GB input moments and applied moments. The panels (**a**–**f**) show the GB input moments $T_{x_{GB}}$, $M_{y_{GB}}$ and $M_{z_{GB}}$ and applied moments $T_{x_{appl}}$, $M_{y_{appl}}$ and $M_{z_{appl}}$, respectively, in a multiple-load test scenario as indicated by the labels of the respective axes.

For the validation of the 6-DOF gearbox input loads, a test scenario with multiple applied loads is employed. This multiple-load test scenario is based on the quasi-statistical design of accelerated load testing as developed by Azzam et al. in [29]. The 3-DOF moments $T_{x_{appl}}$, $M_{y_{appl}}$ and $M_{z_{appl}}$ applied by the aforementioned load application system are shown side-by-side to the 3-DOF GB input loads $T_{x_{GB}}$, $M_{y_{GB}}$ and $M_{z_{GB}}$ estimated in the abovementioned multiple-load test scenario are shown in Figure 9.

**Figure 10:**
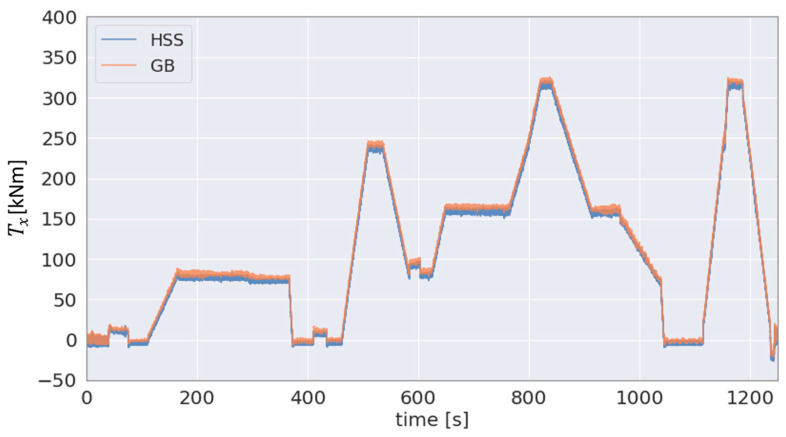
Estimated GB input torque at and measured torque at HSS (scaled using GB ratio).

The GB input torque estimated according to the methodology outlined in Section 2.1 is shown side by side with the applied torque in Figure 9a,b, respectively. The GB input bending moment $M_{y_{GB}}$ computed according to the methodology outlined in Section 2.1 and the applied bending moment $M_{y_{appl}}$ are shown in Figure 9c,d, respectively, for comparison. In addition, Figure 10 can be used to compare the estimated torque using the proposed method ($T_{x_{GB}}$) to the directly measured torque at the HSS ($T_{x_{GB_{HSS}}}$). For a more quantitative comparison between the two variables shown in the figure, the *R*^2^ score is above 0.99. 

**Figure 11:**
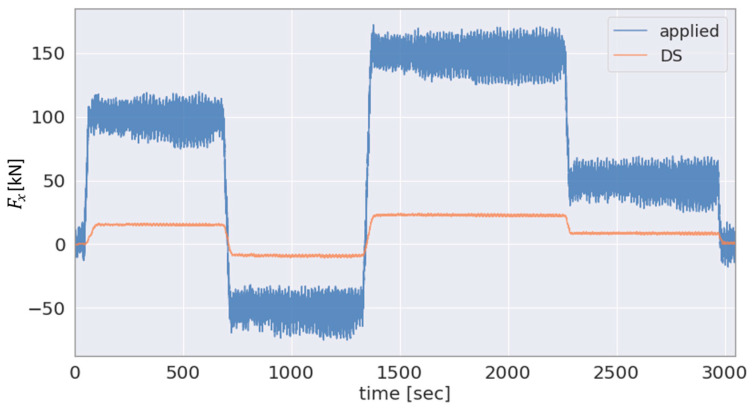
Applied and DS-based estimation in the single-load test scenario for the force $F_{x}$.

**Figure 12:**
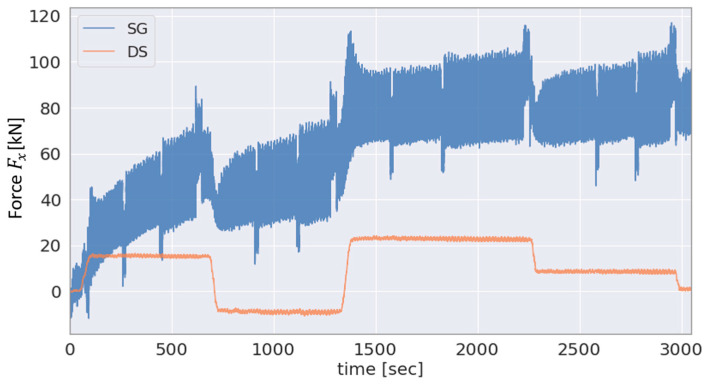
SG-based and DS-based GB input axial force, $F_{x_{GB}}$ and $F_{x_{DS}}$ respectively, in the single-load test scenario for the force $F_{x}$.

To select the most effective approach for the estimation of the GB input axial force $F_{x_{GB}}$ from among the different solutions (see Section 2.3), the axial force based on the different sensor data is provided in Figure 11 and Figure 12. Figure 11 shows the applied and DS-based axial force and Figure 12 the SG-based GB input axial force and the DS-based axial force in the single-load test scenario for the force $F_{x}$. 

**Figure 13:**
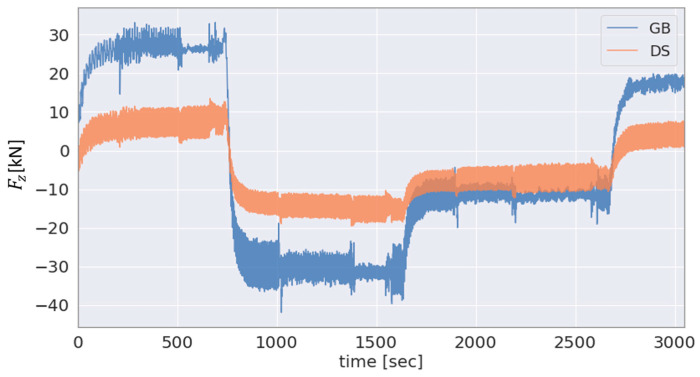
SG-based GB input force $F_{z_{GB}}$ and DS-based $F_{z_{DS}}$ in the single-load test scenario for the force $F_{z}$.

Figure 13 shows the SG-based GB input force $F_{z_{GB}}$ and the DS-based force $F_{z_{DS}}$ the ratio of which is used as a scale for the GB input forces $F_{y_{GB}}$ and $F_{z_{GB}}$, as is explained in Section 2.3.

**Figure 14:**
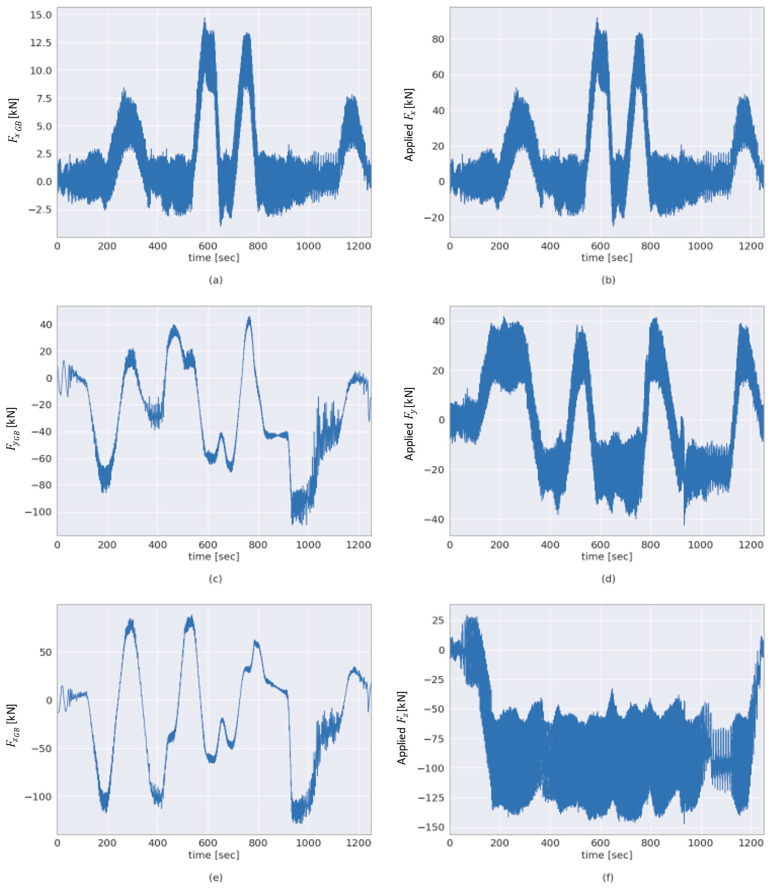
Comparison between estimated GB input forces and applied forces. The panels (**a**–**f**) show the GB input forces $F_{x_{GB}}$, $F_{y_{GB}}$ and $F_{z_{GB}}$ and applied forces $F_{x_{appl}}$, $F_{y_{appl}}$ and $F_{z_{appl}}$, respectively, in a multiple-load test scenario as indicated by the labels of the respective axes.

Furthermore, the 3-DOF forces $F_{x_{appl}}$, $F_{y_{appl}}$ and $F_{z_{appl}}$ applied and the estimated 3-DOF GB input loads $F_{x_{GB}}$, $F_{y_{GB}}$ and $F_{z_{GB}}$ in the aforementioned multiple-load test scenario are shown in Figure 14. Figure 14a shows the GB input axial force of the alternative, hybrid approach outlined in Section 2.3 next to the applied axial force in Figure 14b. For comparison, Figure 14c,e show the GB input forces $F_{y_{GB}}$ and $F_{z_{GB}}$ estimated according to the methodology outlined in Section 2.1 side by side with the applied forces $F_{y_{appl}}$ and $F_{z_{appl}}$, depicted in Figure 14d,f, respectively. The results provided in this section are discussed in the following section.

## 4. Discussion

For the first application of the proposed methodology outlined in Section 2.1, a multiple-load test scenario covering different levels of loads and combinations thereof is utilized. The proposed method is also applied on single-load test scenarios where all loads except for the load of interest are kept constant at the unloaded state of a WT. The results of this application demonstrate the capability of the proposed methodology to estimate the 6-DOF GB input loads. 

As can be seen in Figure 9, the 3-DOF GB input moments, $T_{x_{GB}}$, $M_{y_{GB}}$ and $M_{z_{GB}}$, show agreement with the corresponding 3-DOF moments $T_{x_{appl}}$, $M_{y_{appl}}$ and $M_{z_{appl}}$ as applied by the load application system of the 4 MW WT test bench. The estimated torque $T_{x_{GB}}$ demonstrates a high correlation with the torque ${T_{x}}_{appl}$ applied by the test bench, as shown in Figure 9a,b, respectively. The aforementioned figures also show that the two torques are of a comparable magnitude. In addition, as outlined in Section 2.2, there are torque measurements on the HSS of the GB $T_{x_{HSS}}$ to validate the GB input torque $T_{x_{GB}}$. In order to visually compare the torque $T_{x_{HSS}}$ to the torque $T_{x_{GB}},$ the HSS torque was scaled up using the GB ratio ($i_{GB}$ = 61.92) in Figure 11. As a result, the range of fluctuation in the scaled up $T_{x_{HSS}}$ is apparently higher in the figure than that of $T_{x_{GB}}$, which is measured at the LSS. Figure 10 demonstrates the strong agreement between the directly measured torque $T_{x_{HSS}}$ and the estimated torque $T_{x_{GB}}$ with the proposed method achieving an R2 score of 0.99+ based on Equation (10). 

Since there is currently no available load measurement solution capable of sensing WT GB input loads in 6-DOF [1,25,26], a qualitative validation is performed by comparing the estimated GB input loads to the loads applied by the test bench. Due to the main bearing arrangement, the two aforementioned sets of loads, GB input loads and the loads applied by the test bench, are expected to differ, as shown previously in Figure 4. As outlined in Section 2.1 and Section 2.2, this is a result of the respective location of measurement of these two sets of loads. For example, this difference is apparent in the estimated GB input and applied bending moments around the *y*-axis, as shown in Figure 9c,d. Likewise, this is the case for the estimated GB input and applied bending moments around the *z*-axis, shown in Figure 9e,f. However, a degree of correlation can be seen between the bending moment $M_{y_{GB}}$ at the GB input and the applied bending moment ${M_{y}}_{appl}$ in Figure 9c,d. In addition, a degree of correlation can be seen between $M_{y_{appl}}$ and the computed force $F_{z_{GB}}$ at the GB input in Figure 9c and Figure 14e, respectively, and between GB input force $F_{y_{GB}}$ and ${M_{z}}_{appl}$, as shown in Figure 9e and Figure 14c, respectively. This is to be expected, as demonstrated by the loading distributions shown in Figure 4. 

As the main bearing arrangement transmits the majority of the axial force applied by the test bench to the machine carrier, Figure 11, as expected, shows a lower magnitude of the DS-based estimated GB input force $F_{x_{DS}}$ compared to the applied axial force $F_{x_{appl}}$. In addition, Figure 12 shows a comparison for the same test scenario, single-load $F_{x}$ test, between the $F_{x_{GB}}$ and $F_{x_{DS}}$. As can be seen in the figure, the SG-based approach is not well conditioned for estimating the GB input axial force which is likely due to the relatively high axial stiffness of the LSS, as previously explained in Section 2.3. On the other hand, the axial force estimated by the DS is demonstrably highly correlated with the applied axial force $F_{x_{appl}}$, as shown in Figure 11. Thus, the hybrid approach employing $F_{x_{appl}}$ and $F_{x_{DS}}$, as proposed in Section 2.3, is selected for estimating the GB input axial force $F_{x_{GB}}$. The results obtained using this hybrid method as well as using the SG-based approach are shown in Figure 12, respectively. As expected, due to the main bearing arrangement, a lower magnitude can be seen in the GB input load $F_{x_{DS}}$ compared to the force $F_{x_{appl}}$ applied by the test bench. 

Since the bushing stiffnesses are known from the tests outlined Section 2.2, the DS-based estimation is used as a reference for the validation of the GB input force $F_{z_{GB}}$ in the case of the single-load test scenario for Fz as shown in Figure 13.As can be seen in the figure, the SG-based $F_{z_{GB}}$ and the DS-based $F_{z_{DS}}$ are proportional to each other, with a magnitude difference between the two variables. More specifically, the SG-based $F_{z_{GB}}$ is roughly double the amplitude of the DS-based $F_{z_{DS}}$ throughout the test run. As discussed previously in Section 2.3, the measured shear strain includes errors such as the deviation in the amplitude of the signal of one of the shear SGs relative to the other two shear SGs leading to amplitude deviations in the computed loads. Since $F_{y_{GB}}$ and $F_{z_{GB}}$ are both derived from the affected shear strain gauges, the deviation in the amplitude shown in Figure 13 likely originates from the aforementioned deviations in the amplitude of the shear strain signals. 

In the first application of the proposed method, a drivetrain based on the Vestas V52 was selected, due to its availability. It can be seen in Figure 11 and Figure 14 that the levels of fluctuation of the loads applied by the test bench are relatively high compared to the set load limits used for testing the V52 drivetrain. Compared to more modern WT drivetrains, the V52 has a relatively low rated power of 850 kW. On the other hand, the 4MW WT system test bench is capable of applying significantly higher loads than the design loads of the V52 drivetrain. Therefore, the deviations in the applied loads shown in Figure 11 and Figure 14 are significantly lower when compared to the rated load limits of the test bench than they are in relation to the rated design loads of the V52 drivetrain.

## 5. Conclusions

In this paper, a methodology for estimating the 6-DOF WT GB input loads during WT drivetrain testing was presented. The aforementioned loads were both qualitatively and quantitatively validated using alternative load sensing techniques, such as direct measurements of the applied test bench loads, distance measurements of components with known stiffness and a torque transducer on the HSS, as well as by employing domain knowledge about the drivetrain design. The main conclusions can be stated as follows:

1. The proposed sensor setup consisting of six full-bridge strain gauges and three 3D accelerometers mounted along the circumference of the LSS can be used in combination with the proposed data processing techniques to reach an unprecedented estimation of the 6-DOF GB input loads during WT testing.
2. Challenges and considerations for the practical implementation of the proposed method were identified after its first application on a three-point bearing suspension drivetrain based on the Vestas V52.
3. Signal processing techniques were developed and presented to mitigate against the identified practical challenges in the first implementation of the proposed method such as noise and variations in the input sensor signals.
4. Due to the promising results achieved using the presented methodology, the authors aim to utilize it in upcoming load investigations of WT drivetrains using a WT system test bench capable of controllably applying 6-DOF emulated rotor loads.

## Figures and Tables

**Table 1:** Estimated loads and their respective attributes.

ID	Subject(s)	Measurement	Axis of Measurement
$T_{x_{GB}}$	GB input load	Moment	*x*
$M_{y_{GB}}$			*y*
$M_{z_{GB}}$			*z*
$F_{x_{GB}}$	GB input load	Force	*x*
$F_{y_{GB}}$			*y*
$F_{z_{GB}}$			*z*

**Table 2:** Sources for the validation of loads.

ID	Subject(s)	Measurement	Axis of Measurement	Source
$F_{x_{appl}}$	Rotor input load	Force	*x*	Test bench
$F_{y_{appl}}$			*y*	
$F_{z_{appl}}$			*z*	
$T_{x_{appl}}$	Rotor input load	Moment	*x*	
$M_{y_{appl}}$			*y*	
$M_{z_{appl}}$			*z*	
$F_{x_{DS}}$	GB input load	Force	*x*	DS and Bushing Stiffness
$F_{z_{DS}}$			*z*	

## Data Availability

The data is not publicly available.

## Nomenclature

**Symbol**	**Unit**	**Description**
$M_{y_{GB}}$	Nm	GB input moment around *y*-axis
$M_{y_{applied}}$	Nm	Applied bending moment around *y*-axis
$M_{z_{GB}}$	Nm	GB input moment around *z*-axis
$M_{z_{applied}}$	Nm	Applied bending moment around *z*-axis
$F_{{x_{DS}}_{}}$	N	DS-based force in *x*-direction
$F_{{z_{DS}}_{}}$	N	DS-based force in *z*-direction
$F_{x_{GB}}$	N	GB input force in *x*-direction
$F_{x_{applied}}$	N	Applied force in *x*-direction by test bench
$F_{y_{GB}}$	N	GB input force in *y*-direction
$F_{y_{applied}}$	N	Applied force in *y*-direction by test bench
$F_{z_{GB}}$	N	GB input force in *z*-direction
$F_{z_{applied}}$	N	Applied force in the *z*-direction by test bench
$I_{y}$	$m^{4}$	Second moment of area with respect to the y-axis
$I_{z}$	$m^{4}$	Second moment of area with respect to the z-axis
$K_{ax}$	−	Coefficient matrix for computing load vector $L_{ax}$
$K_{sh}$	−	Coefficient matrix for computing load vector $L_{sh}$
$L_{ax}$	−	Vector of the loads computed with axial SG
$L_{sh}$	−	Load vector computed using shear SG
$T_{x_{GB_{i}}}$	Nm	GB input torque at each time step *i*
$T_{x_{GB}}$	Nm	GB input torque
$T_{x_{HSS_{i}}}$	Nm	GB torque (HSS) at each time step *i*
$T_{x_{HSS}}$	Nm	GB torque (HSS)
$T_{x_{applied}}$	Nm	Applied torque
$W_{T}$	$m^{3}$	Torsional section modulus
${acc}_{y_{i}}$	g	Acceleration in *y*-direction measured by the *i*-th sensor set on the LSS
${acc}_{z_{i}}$	g	Acceleration in *z*-direction measured by the *i*-th sensor set on the LSS
$i_{GB}$	−	GB ratio
$m_{corrected}$	−	Slope of the corrected strain signal after applying Pseudocode in Algorithm 1
$m_{original}$	−	Slope of the original strain signal
$r_{in}$	m	Inner radius of LSS
$r_{out}$	m	Outer radius of LSS
$t_{\varepsilon }$	−	Indices of the window for Pseudocode in Algorithm 2
$\varepsilon_{rotated}$	−	Strain signal of the window after applying Algorithm 2
$\epsilon_{By}$	−	Maximum axial strain induced by the bending moment $M_{y}$
$\epsilon_{Bz}$	−	Maximum axial strain induced by the bending moment $M_{z}$
$\epsilon_{N}$	−	Axial strain induced by normal force
$\epsilon_{ax}$	−	Axial strain vector
$\epsilon_{i}$	−	Strain measured from axial SG connected to the *i*-th telemetry module
$\phi_{i}$	−	Angular position of *i*-th telemetry module
f	Hz	Sampling rate of the used Savitzky-Golay filter
ϴ	−	Angle between the two gradients for Pseudocode in Algorithm 2
ε	−	Strain signal of the window after applying Pseudocode in Algorithm 1
τ	$\frac{kg}{ms^{2}}$	Shear stress
$\tau_{T}$	$\frac{kg}{ms^{2}}$	Shear stress resulting from torsional load
T	Nm	Torsional load
$\tau_{F_{i}}$	$\frac{kg}{ms^{2}}$	Shear stress resulting from later force in the *i*-direction
$F_{i}$	N	Lateral force in the *i*-direction
$S_{j}$	$m^{3}$	First moment of area with respect to the *j*-axis
$I_{j}$	$m^{4}$	Second moment with respect to the *j*-axis
$b_{i}$	m	Width along the *i*-axis of a beam
$A_{LSS}$	$m^{4}$	Cross-section of LSS
